# A Review of Rock Bolt Monitoring Using Smart Sensors

**DOI:** 10.3390/s17040776

**Published:** 2017-04-05

**Authors:** Gangbing Song, Weijie Li, Bo Wang, Siu Chun Michael Ho

**Affiliations:** 1 Department of Mechanical Engineering, University of Houston, Houston, TX 77004, USA; gsong@uh.edu (G.S.); wli27@uh.edu (W.L.); siuchun.ho@gmail.com (S.H.M.H.); 2 Key Laboratory of Transportation Tunnel Engineering, Ministry of Education, Southwest Jiaotong University, Chengdu 610031, China

**Keywords:** rock bolts, smart sensors, piezoelectric sensors, fiber optic sensors, non-destructive testing

## Abstract

Rock bolts have been widely used as rock reinforcing members in underground coal mine roadways and tunnels. Failures of rock bolts occur as a result of overloading, corrosion, seismic burst and bad grouting, leading to catastrophic economic and personnel losses. Monitoring the health condition of the rock bolts plays an important role in ensuring the safe operation of underground mines. This work presents a brief introduction on the types of rock bolts followed by a comprehensive review of rock bolt monitoring using smart sensors. Smart sensors that are used to assess rock bolt integrity are reviewed to provide a firm perception of the application of smart sensors for enhanced performance and reliability of rock bolts. The most widely used smart sensors for rock bolt monitoring are the piezoelectric sensors and the fiber optic sensors. The methodologies and principles of these smart sensors are reviewed from the point of view of rock bolt integrity monitoring. The applications of smart sensors in monitoring the critical status of rock bolts, such as the axial force, corrosion occurrence, grout quality and resin delamination, are highlighted. In addition, several prototypes or commercially available smart rock bolt devices are also introduced.

## 1. Introduction

Millions of rock bolts are being installed worldwide every year and the consumption of rock bolts continues to increase. In 2011, the global usage of rock bolts was in excess of 500 million bolts and the US accounted for 95 million of those bolts. Rock bolts are rock reinforcing members used to reinforce the unstable rock strata by which subsequent deformation can be resisted. As an important component of the supporting systems, rock bolts have been extensively used to support and stabilize engineering structures, such as slopes, retaining walls, tunnels, bridge abutments, dam foundations, and underground excavations [1]. Rock bolts can be divided into three major types based on their anchoring mechanisms [2]. The first type is the mechanically anchored rock bolt that can be anchored by a slit and wedge mechanism or an expansion shell. The second type is the friction anchored rock bolt, such as split-set and swellex, which are anchored by friction between the bolt and the surrounding rock. The third type is the partially or fully grouted rock bolts that are anchored by cement or resin. The anchorage strength of the partially and fully grouted rock bolts is dependent on the grout strength, the shear resistance at the bolt–grout and grout–rock interfaces.

Accidents due to roof falls (i.e., collapse of the roof) are commonly faced problems in the underground coal mining industry. Statistical data show that roof falls have historically been responsible for approximately 50% of all underground fatalities in US, with nearly 45,000 coal miners killed due to roof falls in the entire history of coal mining [3]. There were 77 fatal accidents and 239 serious accidents caused by roof and side falls in India for the period from 2004 to 2006 [4]. In South Africa, 1087 ground fall accidents were reported in 2006 with 85 people were killed in these accidents [5]. The roof falls can cause injuries, disabilities or fatalities for workers while also detrimentally affecting the mining company due to downtime, interruptions in the mining operations, and equipment breakdown [6]. All of these large roof collapses are closely related to the failure of the roof bolting system. Recent decades have seen a substantial reduction in the number of roof fall accidents due to the worldwide adoption of rock bolts. Rock bolts play an important role in maintaining the integrity of the mining tunnels and reducing roof fall accidents. Attention is therefore focused on the condition monitoring of rock bolts in service.

There are various problems associated with the integrity of rock bolts. The loading status, anchorage quality, and the bolt integrity are of most concern. Exposed to the corrosive groundwater, one of the primary causes of rock bolt failure is rusting or corrosion. Studies showed that in the United Kingdom, most of the rock bolt failures were found to be initiated by pitting corrosion, while in Australia, the fractures of rock bolts were attributed to a combination of bending and stress corrosion cracking [7]. The corrosion of rock bolts can be mitigated by filling the gap between the rock bolt and the rock with cement or grout. For resin grouted rock bolts, their efficiency largely depends on the grout quality which is affected by many factors. Poor grout quality due to over-spinning, incomplete mixing and partially encapsulated bolts is a common issue. For example, it had been reported that approximately half of the fully grouted bolts were not as effective as they were supposed to be for the period from 1996 to 1998 [5,8]. The installation quality and the grouting of resin anchored bolts is therefore considered as a critical issue by the underground mining industry.

Various methods have been developed to determine the integrity of the rock bolt and a conventional approach to address this issue is the pull-out test. However, it is a time-consuming and destructive process. Therefore, the need to develop non-destructive test methods that can be used to determine the integrity of the rock bolt in situ has never been this urgent. Advances in smart sensing materials have provided various potential sensing methods; piezoelectric materials and fiber optic sensors are two of the most widely adopted sensing techniques. In this paper, the methodologies of these smart sensors are critically reviewed from the point of view of rock bolt monitoring. The developments and applications of the smart sensors in monitoring the critical status of rock bolts, such as the axial load, corrosion occurrence, grout inadequacy and resin delamination, are highlighted. Additionally, several prototypes or commercially available smart rock bolt devices are also introduced.

## 2. Basics and Types of Rock Bolts

Rock bolts can be divided into four major types according to the coupling mechanism between the rock bolt and the rock mass, and their functions [9]:

- Mechanically anchored rock bolts;
- Friction-anchored rock bolts;
- Fully grouted rock bolts;
- Instrumented rock bolts.

Each bolt has its own advantages and purpose. A detailed overview of the bolts is provided in the following section.

### 2.1. Mechanically Anchored Rock Bolts

**Figure 1:**
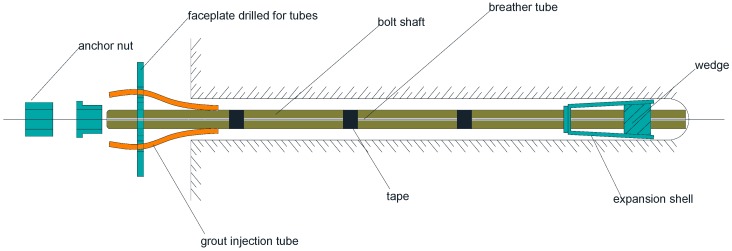
Components of an expansion shell type mechanically anchored rock bolt with provision for grouting.

Mechanically anchored rock bolts are the oldest way of reinforcing underground mining tunnels due to their fast rate of installation. They can be further divided into two types: the slit/wedge rock bolt, and the expansion shell anchor. An example of the expansion shell rock bolt is shown in Figure 1. The main components are a bolt shaft, a faceplate, a conical expansion shell and a wedge. The expansion shell and the wedge are screwed onto the bolt shaft and the entire assembly is inserted into the drilled hole on the rock mass [10]. Rotating the end of the bolt will force the shell to expand against the rock wall of the hole, thereby increasing the anchor force. The anchoring force of this type of rock bolt mainly comes from the friction and interlocking forces between the expansion shell and the wall of the borehole. The anchoring force of this type of bolt is relatively low because the effective anchoring length of the expansion shell is very limited. The bolt is generally tensioned to approximately 70% of the ultimate bearing capacity of the bolt after being installed initially. The pretension gives a reserving capacity of the bolt in case of additional load induced by displacement of the rock mass. 

Mechanically anchored rock bolts perform better in hard rocks but are not very effective in soft rocks due to the large deformation and breakage of the rock in contact with the wedge grips. This type of bolt should be used in rock strata that has a uniaxial compressive strength of more than 50 MPa [11]. The mechanically anchored rock bolts have several advantages in terms of low cost, and rapid installation. However, their effectiveness can be reduced in corrosive environments, where the corrosion of the rock bolt is the primary cause of rock bolt failure. In such case, grouting of the rock bolts is used to protect the rock bolts from the corrosive environments. Another disadvantage of this type of rock bolt is that they will lose their load bearing capacity completely if any slip occurs or the bolt breaks. While this is not a problem for other types of rock bolts. Therefore, they are mainly used for temporary reinforcement or in combination with later grouting or corrosion protection.

### 2.2. Friction Anchored Bolts

Friction anchored rock bolts work by the frictional resistance generated from the bolt/rock interface along the whole bolt length [10]. Since the load transfer mechanism is dependent on the friction force, this type of bolt does not require any important equipment such as the mechanical interlocking device or grouting to transfer the load to the reinforcement. Based on the mode of operation, the frictional bolts can be grouped into two types: Swellex and Split Set.

**Figure 2:**
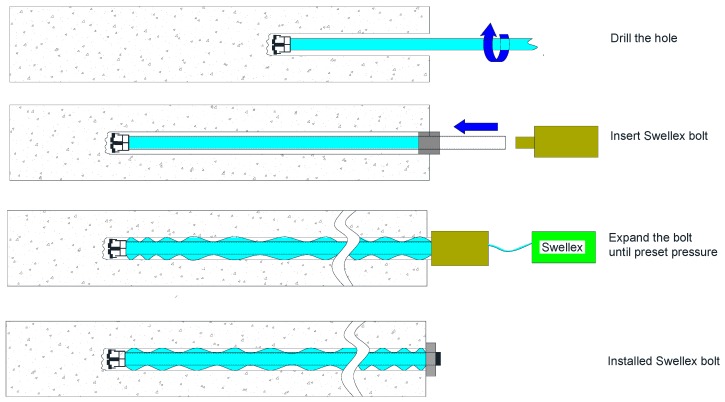
The installation of a Swellex frictional anchored rock bolt.

The Swellex bolt, which was developed and marketed by Atlas Copco, consists of a tube which is folded during the manufacturing process to create a smaller diameter unit. The installation procedure of Swellex bolts is shown in Figure 2. When the bolt is set in place in the borehole, the folded bolt is expanded by hydraulic pressure of approximately 30 MPa, which produces a tight frictional contact and mechanical interlocking at the bolt/rock interface [12]. Immediate support is obtained after installation. 

The Split Set rock bolts were originally developed by James Scott and were commercialized by Ingersoll-Rand Company [12]. The Split Set rock bolts rely solely on friction between the outer surface of the bolt and the rock mass [13]. The Split Set rock bolts consist of a slotted, hollow, high-strength steel tube and a faceplate. The bolt, with greater diameter than that of the borehole, is pushed into the slightly undersized hole. Therefore, a radial spring force is created by the compression of the C-shaped steel tube and a tight frictional contact at the bolt/rock interface is generated.

These frictional anchored rock bolts have the advantages of simplicity, rapid speed of installation and immediate support [14]. Another important property of this type of bolt is that they are able to accommodate large rock deformation. However, their load bearing capacity is low in general [15]. On the other hand, corrosion of these types of rock bolts is a major issue since the bolts are directly exposed to the aggressive environments. Therefore, they cannot be used in long-term applications in corrosive environments.

### 2.3. Fully Grouted Rock Bolts

The working principle of fully grouted rock bolts is that the whole length of the bolt is bonded continuously to the rock mass through resin or cement. Therefore, it can be classified into two types: resin-grouted rock bolts and cement-grouted rock bolts [16]. This type of bolt normally consists of three components: a reinforcing bar, an anchorage faceplate, and the bonding material. The surface of the reinforcing bar is generally in a deformed or ribbed profile to improve the bonding efficiency between the rebar and the bonding agent. Fully grouted bolts can be advantageously used in long-term and systematic applications as grouting agent protects the bolt from the corrosive underground waters. 

**Figure 3:**
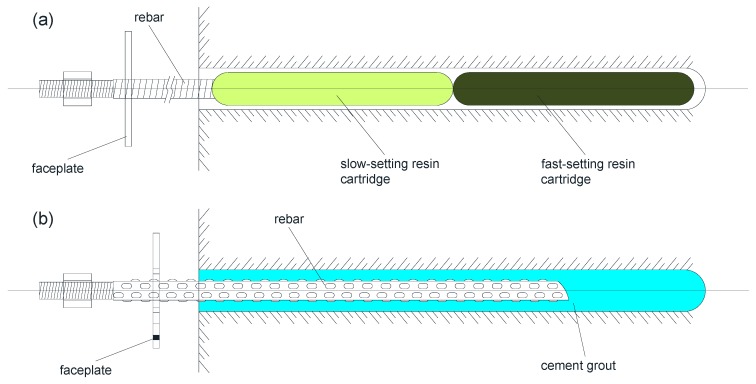
Fully grouted rock bolt: (**a**) fully resin-grouted rock bolt and (**b**) fully cement-grouted rock bolt.

The fully resin-grouted bolts are the most popular type of rock bolting system. A resin cartridge contains a certain amount of resin and a catalyst in separate compartments. As shown in Figure 3a, the cartridges are first set in place in the borehole and then the bolt shaft is rotated into the resin cartridges. The rotation of the bolt breaks the plastic sheath of the cartridges and then mixes the resin and catalyst together [12]. Two different resin cartridges are used together: the fast-setting resin cartridge which is placed further inside the borehole and the slow-setting one which is placed behind the fast-setting one. The former provides instant setting before tensioning of the bolt, which takes only two to three minutes. The latter sets the bolt in place in just 30 min. The fast setting of the resin bonding agent ensures the fast installation as compared to the cement-grouted rock bolts. However, in weak argillaceous rocks or in highly fractured rocks, good bonding between the bolt and the rock mass is difficult to achieve. In such cases, cement grout is considered despite it taking a longer time to set.

Grouting with cement is an inexpensive and traditional way to provide bonding along the full length of the bolt. As shown in Figure 3b, when the bolt is set in place, the cement grout is pumped into the borehole through the hole in the rebar center or a plastic tube alongside the rebar. The cement-grouted rock bolt is always the primary choice for rock reinforcement when it comes to weak or fractured strata. Portland cement is adequate for most applications. The fully cement-grouted rock bolts have the disadvantages of cement shrinkage which reduces the bonding strength, and longer setting time of the cement [16,17]. 

### 2.4. Instrumented Rock Bolts

**Figure 4:**
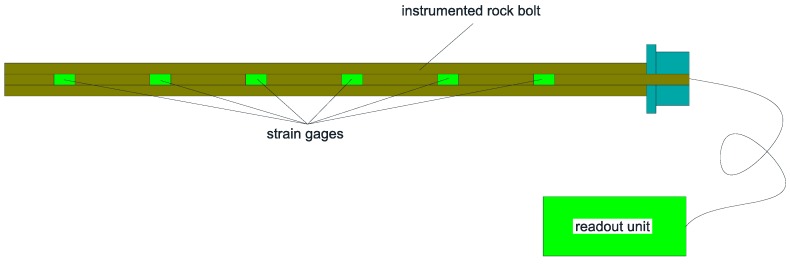
The illustration of an instrumented rock bolt.

Instrumented rock bolts are a special type of bolts that are instrumented with strain gages within the bolt, which are used to monitor the axial force and the strain distribution along the length of the bolt. As illustrated in Figure 4, an instrumented rock bolt generally consists of a bolt shaft, several spaced strain gages along the axis of the bolt, and a portable readout unit for the strain gages. The installation of instrumented rock bolts is no different to the conventional rock bolts. It is very important to ensure that all the strain gages are located within the loaded portion of the rock bolt in order to obtain accurate loading results.

The instrumented rock bolts play an important role in providing useful warning information to mining workers on the impending collapse of the strata. A proper arrangement of the instrumented rock bolts will give the loading capacity distribution of the strata. Based on such information, a wider bolting system can be adopted while maintaining the safety requirements and thus reducing the equipment cost significantly.

## 3. Related Smart Sensors

### 3.1. Piezoelectric Sensors

Piezoelectricity is the phenomenon that a piezoelectric material is able to convert mechanical energy into electrical energy and vice versa. In a direct manner, electric charges are generated when the piezoelectric material is mechanically stressed. Conversely, the geometry of the piezoelectric material will deform according to the applied electrical field. The direct effect of the piezoelectric material can be utilized in dynamic sensing applications. Furthermore, the converse effect can be used in actuation [18]. There are a few materials that possess the properties of piezoelectricity, including piezoelectric ceramics (Lead Zirconate Titanate, also known as PZT), piezoelectric polymers (Polyvinylidene Fluoride, denoted as PVDF) and piezoelectric ceramic/polymer composites, which are frequently used as piezoelectric actuators and sensors for structural health monitoring of various structures [19,20]. 

According to compact matrix notation [21], the coupled electromechanical constitutive equations of a linear piezoelectric material can be expressed as

(1) $$\left[\begin{array}{c} D\\ S \end{array}\right]=\left[\begin{array}{c} \varepsilon^{T}\;d\\ d^{t}\;s^{E} \end{array}\right]\left[\begin{array}{c} E\\ T \end{array}\right]$$

where D and E are the electric displacement (3 × 1) (C/m^2^) and electric field (3 × 1) (V/m) respectively. S and T are the mechanical strain (6 × 1) and stress (6 × 1) (N/m^2^), d, $\varepsilon^{T}$ and $s^{E}$ are the piezoelectric strain constant (3 × 6) (C/N or m/V), dielectric permittivity (3 × 3) (Farad/m) and compliance constant (6 × 6) (m^2^/N), respectively. When the mechanical stress/strain are in the x-direction and the electrical field/displacement are in the z-direction, the constitutive equations can be simplified as

(2) $$D_{3}=\varepsilon_{33}^{T}E_{3}+d_{31}T_{1}\;S_{1}\;=s_{11}^{E}T_{1}+d_{31}E_{3}$$

Various non-destructive testing (NDT) techniques based on piezoelectricity have been developed over the last decades, such as Lamb wave [22,23], electro-mechanical impedance (EMI) [24,25,26], guided ultrasonic waves [27], and acoustic emission (AE) [28,29]. Lamb waves are a form of elastic perturbation that can propagate in a solid plate with free boundaries, which can be excited by piezoelectric patches driven at a particular frequency. The time of flight, propagation velocity, amplitude and phase of Lamb waves can be utilized to locate, detect and quantify a damage in a plate structure. The electro-mechanical impedance technique measures the changes in the frequency response of the electrical impedance of the PZT due to the changes in the mechanical impedance of the host structure. Compared to a baseline measurement in pristine state, the peak frequency shift and amplitude change of the impedance spectra indicate the damage severity of the host structure. The guided ultrasonic waves technique exploits the waveguide geometry of the tested structure, such as planar and tubular structures. The guided ultrasonic waves are excited in this type of structure, which is then reflected from defects such as crack, delamination and corrosion. The location of the defects can be determined from the arrival time of the reflected waves. Unlike Lamb wave, electro-mechanical impedance and guided ultrasonic wave techniques, which belong to active NDT methods, the acoustic emission method is a passive technique. The acoustic emission technique relies on the stress waves generated from the rapid release of energy from a structure due to damage-related deformation. The descriptive parameters of the AE waves offer rich damage-related information, which can be used to characterize the location, source, and evolution of the damage. Among all these piezoelectric-based NDT methods, the guided ultrasonic waves are more suited for condition monitoring of rock bolts since rock bolts act as a waveguide by nature.

#### Guided Ultrasonic Waves in a Cylinder

Guided waves occur only in plate-like or waveguide-like structures, such as plates and bars. Their energy is concentrated near the boundaries. Since they are confined in the waveguide, guided waves are able to propagate over long distance. Usually, the guided waves are excited using a short duration toneburst. In general, the guided waves are dispersive, and their traveling velocity is a function of frequency. Also, multiple modes exist for a specific waveguide. 

**Figure 5:**
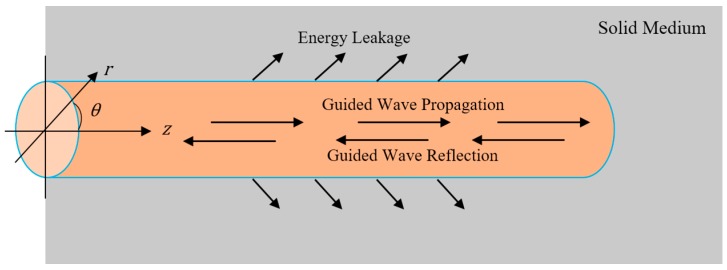
Geometry of a steel bar in solid medium.

The geometry of a steel bar embedded in solid medium is presented in Figure 5. There are three modes in this cylindrical steel bar: the longitudinal modes (0, *m*), torsional modes *T*(0, *m*), and flexural modes *F*(*n*, *m*). Here, *n* and *m* are the circumferential order and modulus, respectively. The selection of a suitable mode for the application of ultrasonic guided waves in NDT is very important; this selection can be made by examining the dispersion curves. The details of the procedure are described in the following references [30,31]. In the guided wave NDT, the arrival time, amplitude, and velocity of the received waves can be used to characterize defects/damage of the waveguide.

### 3.2. Fiber Optic Sensors

Fiber optic sensors are a category of sensors that use the optical fiber or the light traveling through the fiber as the sensing element. Properties of the light will change due to external perturbations such as strain, pressure, temperature and electric currents experienced by the fiber optic. An optical fiber sensor system typically consists of a light source, a receiver, an optical fiber as the sensing element, a modulator, and a signal processing unit. The fiber optic sensors convert or encode these external perturbations into corresponding variations in the optical properties of the transmitted light, such as intensity, phase, wavelength, frequency and polarization [32]. Such variations are then demodulated by a dedicated demodulation system. Fiber optic sensors have several distinctive advantages over conventional electrical sensors which include small size, high sensitivity, corrosion resistance, immunity to electromagnetic interference, and ability to multiplex. Therefore, fiber optic sensors have been widely used in monitoring various engineering structures. Among the various types of fiber optic sensors, the fiber Bragg grating (FBG) sensors and Brillouin optical time domain reflectometer (BOTDR) sensors have shown the most promise in the field of structural health monitoring.

#### 3.2.1. FBG Sensors

A FBG is produced by inscribing periodic and permanent modifications in the core refractive index along the optical fiber axis [33]. When a broadband light is launched through the gratings, the reflected Bragg wavelength follows the form

(3) $$\lambda_{B}=2n_{eff}\Lambda$$

where $\lambda_{B}$ is the Bragg wavelength, $n_{eff}$ is the effective refractive index of FBG and Λ is the grating period. The grating period, and therefore the Bragg wavelength, are linear to both strain and temperature. The relation between the relative Bragg wavelength shift and the axial strain ε and the temperature variation ΔT is expressed as follow

(4) $$\frac{\Delta \lambda_{B}}{\lambda_{B}}=C_{\varepsilon }\varepsilon +C_{T}\Delta T$$

where $C_{\varepsilon }$ and $C_{T}$ are strain and temperature sensitivity coefficients, respectively.

**Figure 6:**
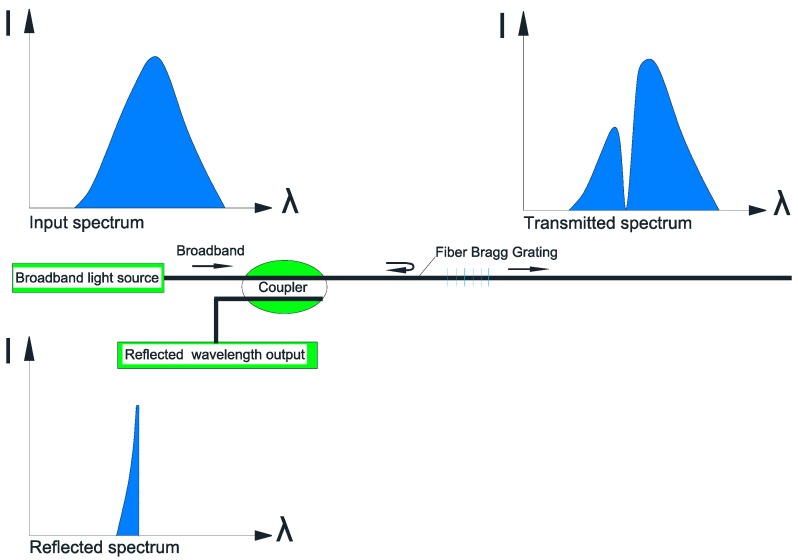
Functioning principle of the FBG sensor.

As illustrated in Figure 6, when a broadband light passes through optical fiber grating, the portion of the spectrum which equals to the Bragg wavelength of the FBG is reflected, and others can pass through the FBG with small attenuation. In this way, the interrogation of FBG sensors can be easily multiplexed using the wavelength division multiplexed (WDM) technique to realize a quasi-distribution sensor network [34].

#### 3.2.2. BOTDR Distributed Sensors

**Figure 7:**
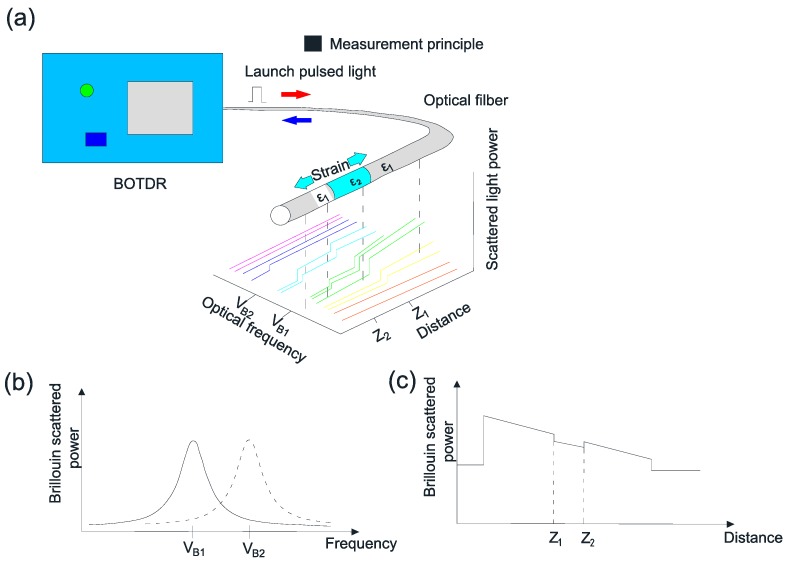
Principle of the Brillouin Scattering-based fiber optic measuring technique: (**a**) The Brillouin scattering spectrum along an optical fiber; (**b**) The Brillouin scattering spectrum at a specific frequency; (**c**) The Brillouin scattering spectrum at a specific location.

Brillouin scattering is a phenomenon in which a light wave transmitted in an optical fiber is scattered by the interaction with acoustic waves [35,36]. The frequency of the scattered Brillouin light is dependent on the strain and temperature of the optical fiber, which will shift from the frequency of the incident light. The Brillouin frequency shift $v_{B}$ is given by the following equation

(5) $$v_{B}=\frac{2nv_{A}}{\lambda }$$

where n is the refractive index, $v_{A}$ is the sound wave velocity and λ is the wavelength of incident light. The Brillouin frequency shift is linearly proportional to the change of strain or temperature, which is expressed as follows

(6) $$v_{B}\left(\varepsilon ,T\right)=v_{B0}\left(\varepsilon_{0},T_{0}\right)+C_{\varepsilon }\left(\varepsilon -\varepsilon_{0}\right)+C_{T}\left(T-T_{0}\right)$$

where $v_{B}\left(\varepsilon ,T\right)$ is the Brillouin frequency shift at strain ε and temperature T; $C_{\varepsilon }$ and $C_{T}$ are the strain and temperature coefficients, respectively; $T_{0}$ and $\varepsilon_{0}$ are the initial strain and initial temperature that correspond to the reference Brillouin frequency $v_{B0}$. Figure 7a shows the Brillouin scattering intensity distribution at different frequencies along the optical fiber. Figure 7b shows the frequency shift of the Brillouin back scattering light at a specific location due to its corresponding strain, Figure 7c shows the Brillouin scattering intensity spectrum at a specific frequency along the optical fiber. BOTDR is very suitable for long-distance distributed strain sensing with a sensitivity of 5 με [37]. However, the spatial resolution is relatively low, about 1 m, and therefore this technique is not suitable for structural monitoring applications that require dense distribution of sensors.

## 4. Rock Bolt Monitoring Using Smart Sensors

### 4.1. Load Monitoring

In order to ensure the proper functioning of rock bolts and that the rock bolts are not subjected to excessive loading, it is important to monitor the load experienced by the rock bolts. The three quantities of primary significance are the applied load at the rock bolts, the bolt head displacement, and the load distribution along the anchored length [1].

#### 4.1.1. Traditional Load Monitoring Methods

**Figure 8:**
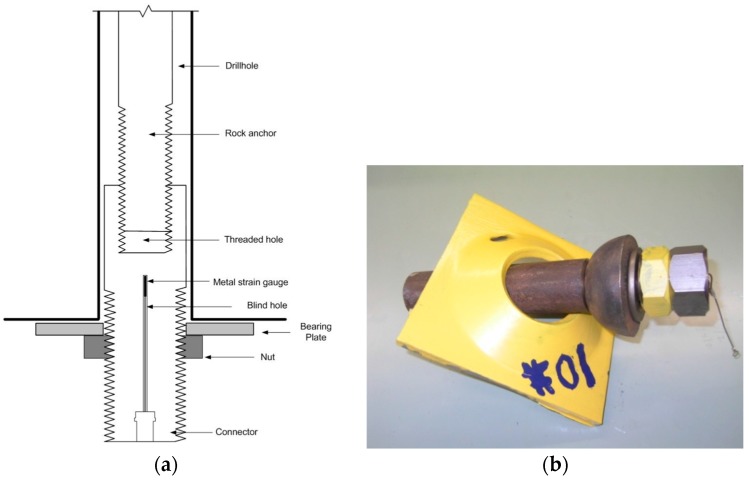
(**a**) The coupler load cell design concept; (**b**) photo of the coupler load cell [38].

One traditional way to measure the rock bolt load was the use of electrical-resistance strain gages, which perform relatively well for short-term load measurement. However, the long-term survivability of electrical-resistance strain gages was still an issue and the gages are prone to damage during and after installation. To compensate for the shortcoming of electrical-resistance strain gages, the vibrating-wire strain gages were developed and commercialized [1,38]. The operating principle of vibrating-wire strain gages relies on the phenomenon in which the natural vibration frequency of a wire changes with its tension. The vibrating-wire strain gages have good stability, long-term reliability and high accuracy and can either be surface-mounted on or be embedded into the structure. Benmokrane et al. investigated the performance of vibrating-wire strain gages instrumented in grouted anchors from the perspectives of the load transfer mechanism, the debonding process, creep behavior, and long-term performance [1]. Compared to the vibrating-wire strain gages, the metal-based strain gages have larger stretch capacity. Therefore, Mitri and Marwan devised a rock bolt load cell by placing a metal strain gage inside the bolt head via a small drilled hole in its center, and implemented such techniques in practical application [39,40]. Mitri later introduced an improved rock bolt load cell based on the concept of the coupler load cell, which has improved performance in terms of measuring the ultimate strength of the rock bolt, shipping, and installation [38]. Figure 8 shows the coupler load cell concept and the photo of the load cell.

#### 4.1.2. Load Monitoring Based on FBG Sensors

**Figure 9:**
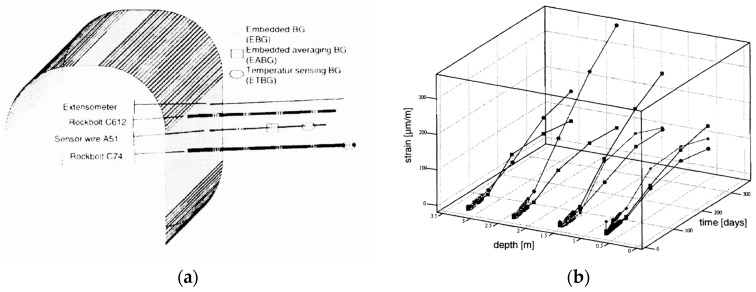
(**a**) Installation of GFRP rock bolts equipped with FBG sensors; (**b**) one year observation of rock movement in the tunnel with the three installed GFRP sensors: wire A51 (diamonds) and rockbolts C612 (squares) and C74 (dots) [43].

Most of the rock bolts on the market are made of steel, but a small portion of rock bolts are made of composite materials such as glass fiber reinforced polymer (GFRP) and carbon fiber reinforced polymer (CFRP). Embedding the fiber optic sensors inside the composite materials is very effective in transferring strain and protecting the fragile optical fiber from mechanical damage. Frank et al. fabricated GFRP rock bolts instrumented with FBG sensors by embedding the FBG sensors in the GFRP rock bolts during the pultrusion process [41]. The tensile tests indicated that the embedded FBG sensors were able to survive a high strain of 1.5%. The GFRP rock bolts equipped with FBG sensors were installed in a tunnel near Sargans, Switzerland for long-term load monitoring [42,43]. The installation scheme of the FBG-equipped GFRP rock bolts in a tunnel and the one year measurements are shown in Figure 9. The collected strain information along the rock bolts reveal the long-term movement of the rock masses and allow operators to take the necessary precaution.

**Figure 10:**
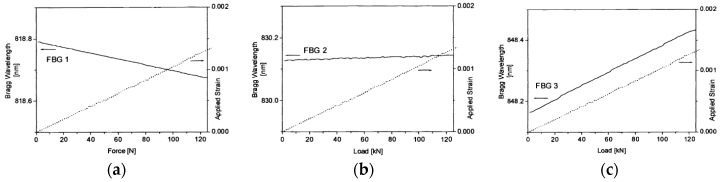
Bragg wavelength change and applied strain vs. load for (**a**) compressed sensor (17°); (**b**) neutral sensor (29°) and (**c**) elongated sensor (40°) [44].

**Figure 11:**
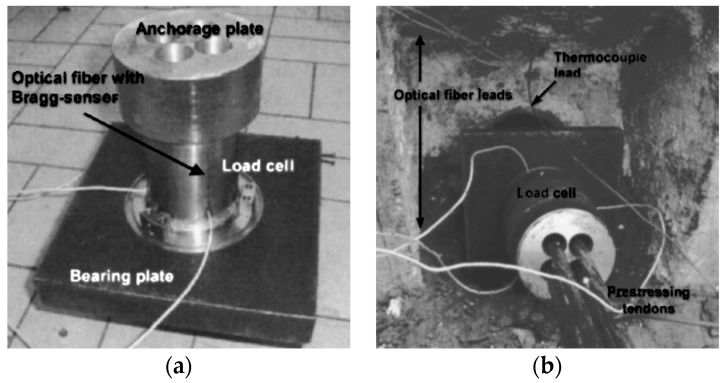
(**a**) The FBG instrumented load cell; (**b**) the load cell is placed in the quay wall [45].

**Figure 12:**
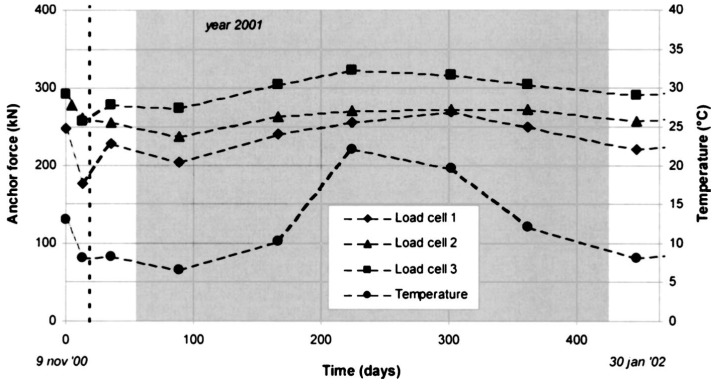
Overview of the monitoring results during a period of more than 1 year after installation of the ground anchors [45].

For steel rock bolts, however, the strain can go up to 20%. A direct mounting of FBG sensors along the bolt shaft can easily damage the sensor under loading condition. Schroeck et al. devised a special arrangement of the FBG sensors in order to measure large relative strain [44]. In their design, they found a neutral line on the cylinder shell, along which the elongation and contraction effects cancel out each other. A FBG sensor bonded on the rock bolt in a small inclination from the neutral angle will give a predictable ratio to the elongation of the rock bolt. Three FBG sensors were installed at 17°, 29° and 40° to the circumferential direction of the bolt to yield strain transfer ratios of −15%, 0% and +15% with respect to the strain of the bolt, respectively. The Bragg wavelength change and applied strain versus the load for the compressed sensor (17°), neutral sensor (29°) and elongated sensor (40°) are illustrated in Figure 10. In this inclination method, it is feasible to measure the large elongation of steel rock bolts without breaking the FBG sensors. Moerman et al. applied the FBG sensors on the load cell, which was placed between the bearing plate and anchorage plate to measure the force in the ground anchor [45]. Each load cell was equipped with three FBG sensors, and the load cell was calibrated to have linear response and an accuracy of 3.5 kN. The FBG-equipped load cells were installed in a quay wall for long-term anchor force monitoring. Figure 11 shows the FBG-instrumented load cell. Figure 12 shows the anchor forces over a 15-month period. 

**Figure 13:**
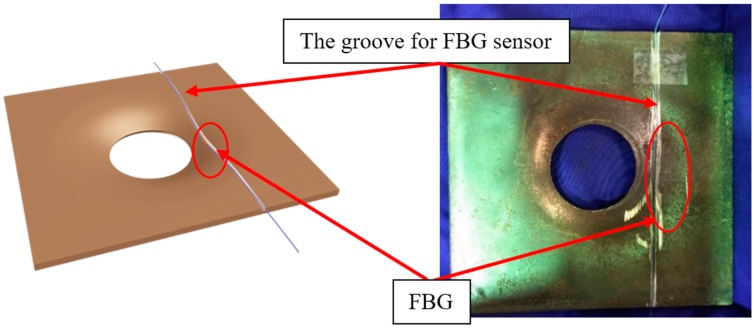
The load measuring anchor plate using an FBG sensor. Note: the circle indicates the location of the FBG strain sensor [46].

**Figure 14:**
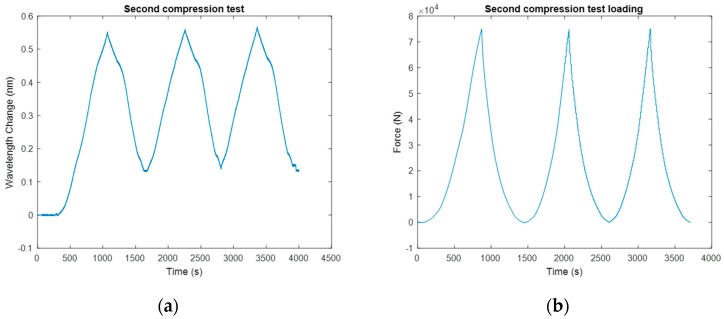
FBG signal for the nondestructive compression test in which the plate was cyclically loaded from 0 to 75 kN: (**a**) Strain; (**b**) Force [46].

Ho et al. instrumented the FBG sensor on the load bearing plate to measure the rock bolt axial load [46]. Figure 13 shows the load measuring anchor plate instrumented with the FBG sensor and the representative testing results are shown in Figure 14. Such a method ensured that the FBG sensor was not over-strained. Other applications of FBG sensors in load monitoring include monitoring the axial stress of rock bolts during the subway tunnel construction using the testing model [47] and investigating the load transfer mechanism of the ground anchor and tensile force distribution along the tendon [48].

#### 4.1.3. Load Monitoring Based on Distributed Brillouin Sensing

**Figure 15:**
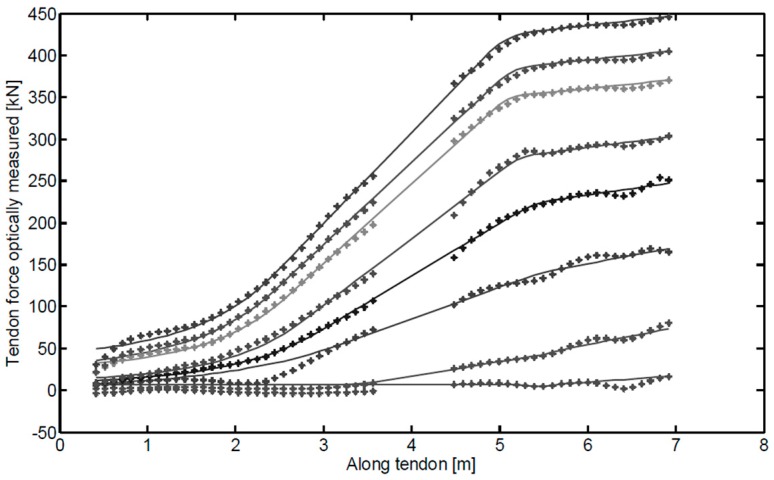
Load distribution measured by the Brillouin scattering sensing technique at different load steps [49].

Unlike the quasi-distributed FBG sensors, the optical fiber Brillouin scattering sensing technique is truly distributed, which can be advantageously used to measure the stress distribution along the rock bolt. Iten and Puzrin examined the Brillouin optical time domain analysis (BOTDA) optical fiber distributed sensing technique in assessing the stress distribution along ground anchors [49]. Three different methods of fiber optic sensor integration into steel tendon—external longitudinal trench, internal integration, and helix integration—were investigated. Among these methods, trench and internal integration methods were more suited for distributed stress monitoring, and therefore they were applied in a wall of an excavation pit for field testing. The load distribution measured by this technique along the tendon for different load steps is shown in Figure 15. The load distribution under different pullout forces was clearly observed using the Brillouin distributed technique. More recently, Moffat et al. experimentally tested the BOTDR technique for rock bolt loading condition monitoring [50].

### 4.2. Corrosion Monitoring

Corrosion is a process involving the physical alternation of a material from a chemical reaction in the corrosive environments which often leads to the reduction of mechanical properties of the material. Steel rock bolts are particularly susceptible to corrosion since they are very likely to be exposed to the corrosive underground water. The rate of steel rock bolt corrosion is dependent on the ground water composition, flow rate, water pH value, temperature, humidity, surface condition, presence of corrosion inhibitors, applied stresses, and any hydrogen sulphide concentrations [51,52]. Stress corrosion cracking (SCC) and pitting corrosion of rock bolts constituted the major cause of premature failure of rock bolts in corrosive environments, where SCC accounted for 63% of the broken bolts and localized pitting corrosion represented 30% [53]. SCC results from the combined effects of high tensile stress and corrosive environment on the susceptible metals, which will result in the progressive development and growth of brittle cracks on the surface of the metal [54]. Localized pitting corrosion is marked by the development of sharply defined holes, or ‘pits’, which result from localized metal loss accelerated by the presence of a small anode and a large cathode [52]. Rock bolt corrosion reduces the load bearing capacity and life expectancy of ground support systems, and therefore the monitoring and determination of rock bolt corrosion is an urgent task. 

**Figure 16:**
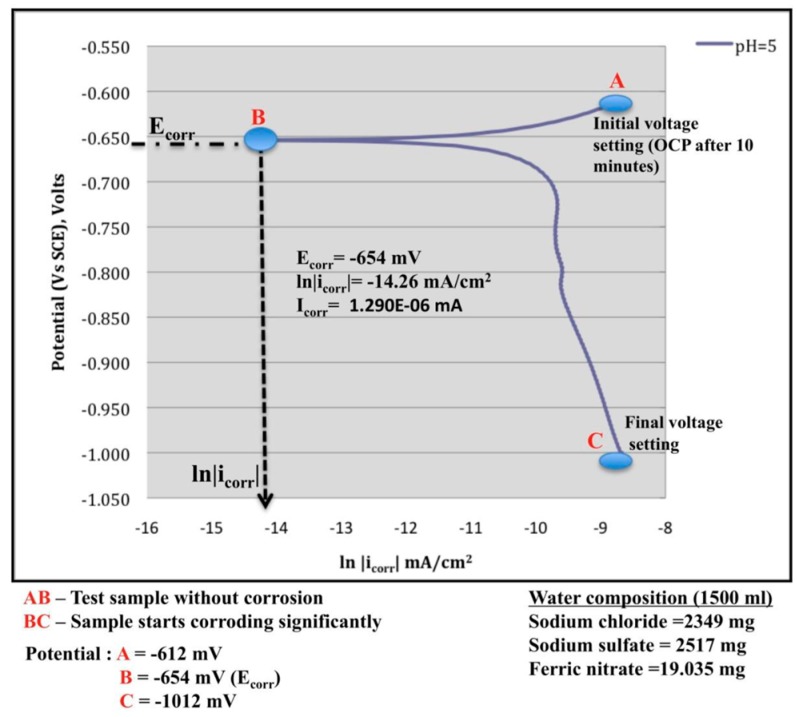
Tefel plot for a test steel sample [55].

Electrochemical-based corrosion measuring methods, such as open circuit potential (OCP) and electrochemical impedance spectroscopy, are the traditional ways to measure corrosion of metals. Spearing et al. [55] used the OCP measurement technique to determine the corrosion potential of rock bolts. As shown in Figure 16, the results showed that a more negative OCP value can indicate the possibility of the presence of corrosion. However, such a method only provides qualitative results.

In order to detect SCC, Craig et al. [53] utilized the ultrasonic crack detector on rock bolts taken from a mine site. A peak will appear in the received signals if there are any signal reflections from cracks or the end of the bolt. The load tests indicated that the bolts with more severe SCC presented stronger reflection signals. 

**Figure 17:**
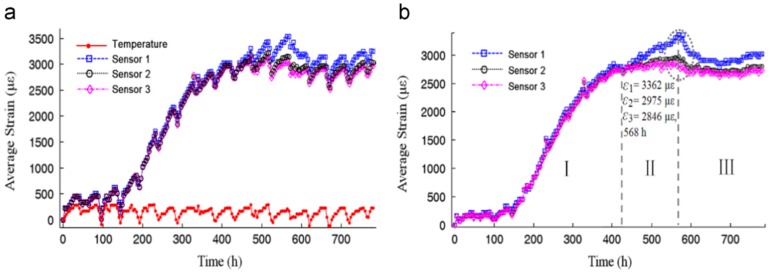
The results from the sensor in R–C–O–C configuration. (**a**) The uncompensated results without the reference fiber; (**b**) The results after being compensated by the reference sensing fiber. [56].

On the account that voluminous corrosion products will exert expansive stress on the surrounding rock mass, Wei et al. [56] proposed a method to monitor rock bolt corrosion in an early stage by measuring the corrosion-induced strain using a fiber-optic Michelson interferometer. Two schemes of winding optical fiber on the rock bolt were tested: one directly wound the fiber on the rock bolt and another one, with a mortar cushion, was placed between the fiber and the rock bolt. The latter scheme showed more uniform strain development under accelerated corrosion experiments, as shown in Figure 17. The test results demonstrated the effectiveness of the proposed sensing technique in monitoring rock bolt corrosion in an early stage.

### 4.3. Grout Quality Testing

The grouted rock bolts, either concrete grouted or resin grouted, account for the majority of the rock bolts. For grouted rock bolts, the grout quality determines the anchorage strength of the bolts. The assessment and control of the grout quality is a challenging issue. The very traditional way to determine the grout quality is by the pull-out test and the over-coring test, which are considered destructive and time-consuming [8,57]. When ultrasonic waves are introduced in structures of slenderness such as rock bolts, guided waves are excited. The use of guided ultrasonic waves for nondestructive monitoring is emerging as a new research initiative in rock bolt testing and has attracted substantial research interest over the last two decades [31,58,59,60,61]. Beard and Lowe [31] pioneered the application of the guided ultrasonic waves in the non-destructive testing of rock bolts. Due to the dispersive nature of the ultrasonic waves, they systematically studied the selection of the suitable test frequencies and identified that the fundamental longitudinal *L*(0, 1) mode was more suited for rock bolt testing. Later on, two research groups, one from Dalhousie University and another one from Korea University, were actively devoted to this research field in recent years. 

**Figure 18:**
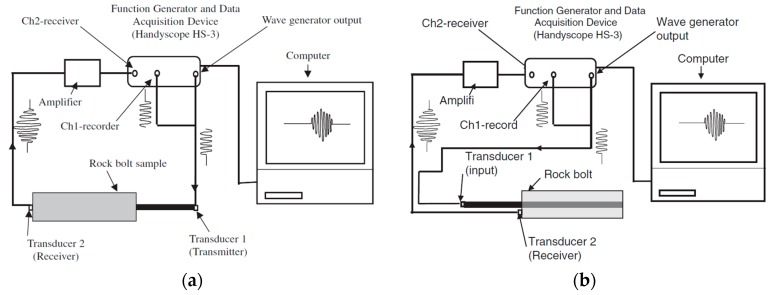
Equipment setup for the rock bolt test: (**a**) transmission-through setup [62]; (**b**) transmission-echo-off-path setup [63].

**Figure 19:**
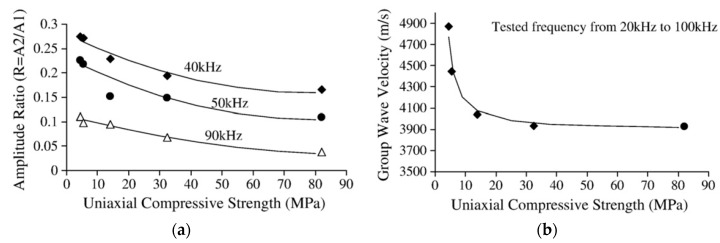
(**a**) Variation of amplitude ratio with grout compressive strength at different wave frequencies; (**b**) Variation of average group velocity with grout compressive strength [60].

The research group at Dalhousie University successively examined the effects of grout strength [58,60], the grouted length [62], and missing grout [61] on the behavior of the ultrasonic guided waves in rock bolts. The instrumentation of the guided ultrasonic waves for rock bolt testing consisted of a function generator and data acquisition device; piezoelectric transducers as the transmitter and receiver; a signal amplifier and personal computer. An example of the equipment setup for transmission-through is shown in Figure 18a. In order to avoid the complexity of multiple modes at higher frequencies and to simplify data interpretation, the *L*(0, 1) mode of low frequencies less than 100 kHz were usually selected. The group velocity and attenuation of the guided ultrasonic waves were the most important characteristics to describe the grout quality. They have concluded that higher grout quality, either longer curing time of the grout or higher compressive strength of the grout, resulted in lower group velocity in grouted bolts and higher attenuation of the waves. The representative results are shown in Figure 19. With the obtained information on the attenuation and group velocity of the guided ultrasonic waves, grout quality and the defective rock bolts can thus be detected. 

The transmission-through setup was generally not practical in the field since only one end of the bolt is exposed for testing. Therefore, they conducted similar tests using the transmission-echo-off-path setup, as shown in Figure 18b [63]. The new setup was shown to be able to receive meaningful results to determine the attenuation and group velocity.

**Figure 20:**
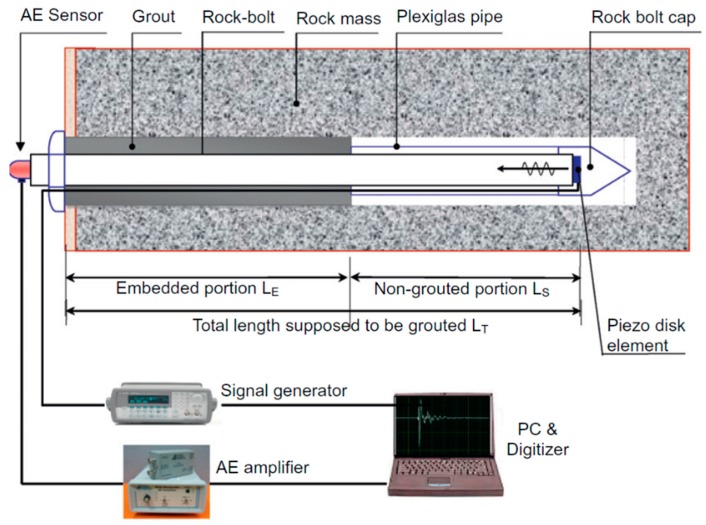
Schematic diagram of the rock bolt integrity system [66].

**Figure 21:**
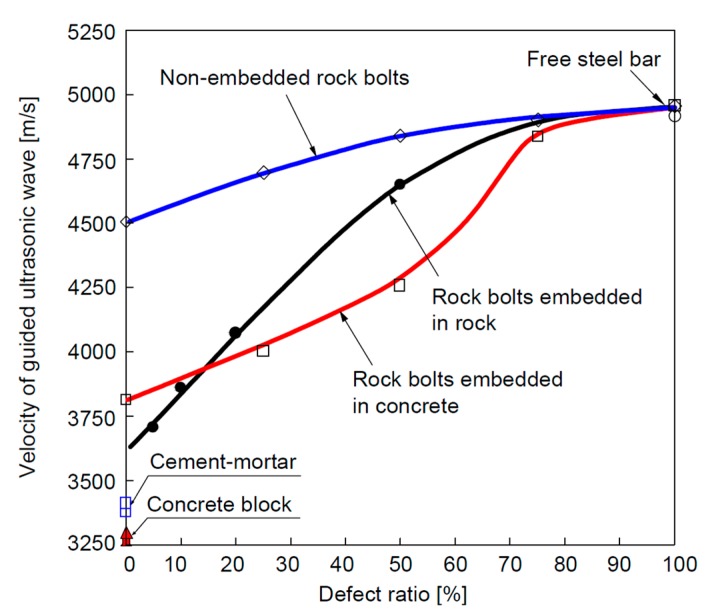
Velocity of guided ultrasonic waves versus the defect ratio (DR) for non-embedded rock bolts, rock bolts embedded in concrete and rock bolts embedded in rock [65]. Note: Velocity of free steel bar *V_S_* = 4955 m/s; velocity of cement mortar *V_M_* = 3336 m/s; velocity of concrete block *V_C_* = 3290 m/s; velocity of rock mass *V_R_* ≥ 2700 m/s.

The research group at Korea University also successively conducted several investigations on the non-destructive evaluation of rock bolt integrity using guided ultrasonic waves, particularly on the non-grouted ratio or defect ratio of the rock bolt [64,65,66,67]. A typical experimental setup of the rock bolt integrity test is shown in Figure 20. Experiments with different defect ratios for grouted non-embedded rock bolts, and for rock bolts embedded into a concrete block and rock mass were carried out. They analyzed the signals in the frequency domain using the Fourier transform and in the time-frequency domain using wavelet transform based on a Gabor wavelet. From the frequency domain analysis, they found that a portion of high frequency content increases with the increase of the defect ratio. The group velocity and phase velocity of the waves were calculated from the peak values in the time-frequency domain analysis. Similar to the findings from the research group at Dalhousie University, this research group concluded that the velocities of the guided ultrasonic waves increased with the increase in the defect ratio in all three conditions, namely non-embedded rock bolts, rock bolts embedded in concrete columns, and rock bolts embedded in a rock mass. The representative results are shown in Figure 21.

The reflection method, in which both the piezoelectric transmitter and receiver were installed at the free end of the bolt, was also examined for practical application [67]. It is worth noting that for the case of rock bolts embedded in a rock mass, the piezoelectric transducer could not generate strong guided waves. The hammer impact with a center punch method was adopted to produce strong reflected guided waves. Therefore, it is suggested that the hammer impact method is more practical to generate the guided waves in field tests.

### 4.4. Delamination Monitoring for Grouted Rock Bolts

**Figure 22:**
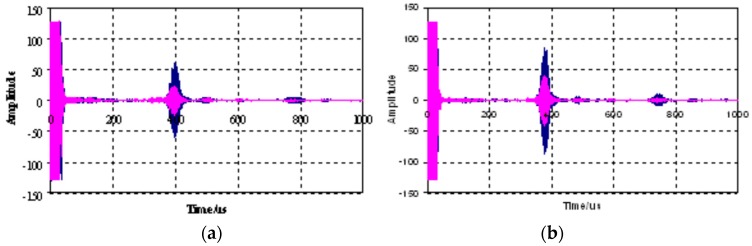
(**a**) Typical waveforms at low frequency modes: 0.97 MHz excitation; (**b**) 1.17 MHz excitation. Pink for no delamination and blue for one-third delamination [68].

Apart from the grout quality monitoring and control, the amount of delamination between the rock bolt and the grout is critical in evaluating the anchorage strength of the rock bolt system. The delamination between the bolt and the grout means a weak bonding condition. He et al. [68] investigated the propagation of guided ultrasonic waves in a rock bolt system theoretically and experimentally. First off, they calculated the high-frequency theoretical wave structures for a steel rock embedded in concrete using the Semi-Analytical Finite Element Method and identified that low frequency waves were suitable for length measurement and delamination detection since these waves were susceptible to leakage at the interface. In the experimental studies, six specimens with different delamination lengths, such as 0%, 25%, 33%, 50%, 70% and 100%, were prepared. They adopted a pulse-echo arrangement using the tone-burst system with piezoelectric transducers. Both high frequency modes (above 2 MHz) and low frequency modes (below 1.6 MHz) were used to excite the guided waves. Results showed that the high frequency modes were not sensitive to the presence of delamination. However, the low frequency modes were more sensitive to the delamination damage, as shown in Figure 22. A gain of approximately 6 dB correlated to one-third delamination.

**Figure 23:**
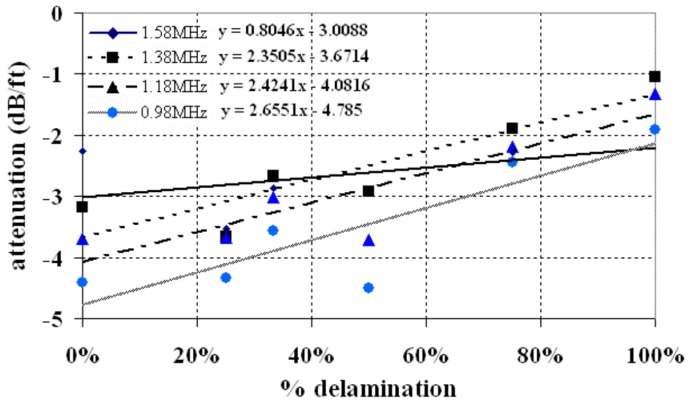
The attenuation versus the delamination percentage [68].

The relationship between attenuation and the delamination percentage is shown in Figure 23. It is clear that the wave amplitude increases with the increase of delamination percentage. The fitted slopes suggested that low frequencies are more sensitive to delaminated damage of the grouted rock bolts. The study concluded that guided waves with low frequency modes were efficient for estimating the amount of delamination between the rock bolt and the grout.

Interfacial debonding and delamination issues are also encountered in many other engineering structures such as composites, and concrete-encased steel structures. Zeng et al. [69] showed that the active sensing approach based on the shear mode of piezoelectric transducers was effective in detecting the interfacial debonding between concrete and steel in a concrete-encased composite structure. It is believed that the active sensing method using shear waves will be promising in detecting the delamination of grouted rock bolts.

### 4.5. Rock Bolt Devices with NDT Capability

Several rock bolt testing system were developed over the last two decades that were specialized for non-destructive testing of rock bolts. Some of them are commercially available and some are undergoing prototyping and testing. 

The Boltometer concept was proposed by Geodynamik in Sweden. The Boltometer uses a piezoelectric transducer to transmit compressional and quasi-flexural waves into the rock bolt and receives the echoes reflected from the discontinuities in the bolt and the grout [5]. The Boltometer is able to indicate bad grout that generates a distinct echo. If the grout quality is good, the energy will be dissipated into the rock mass, leaving little or no echoes.

The ground anchorage integrity testing (GRANIT) system was developed at University of Aberdeen in Scotland [70]. The system induces low frequency vibrations to the rock bolt by an impact device and receives the vibration signals by an accelerometer. The acceleration signals are complex by nature. Therefore, neural networks were used to interpret the relationship between bolt condition and the response signals. The need for training neural networks to recognize existing conditions means that the system cannot be easily applied for new rock bolt conditions that have never been characterized before. 

**Figure 24:**
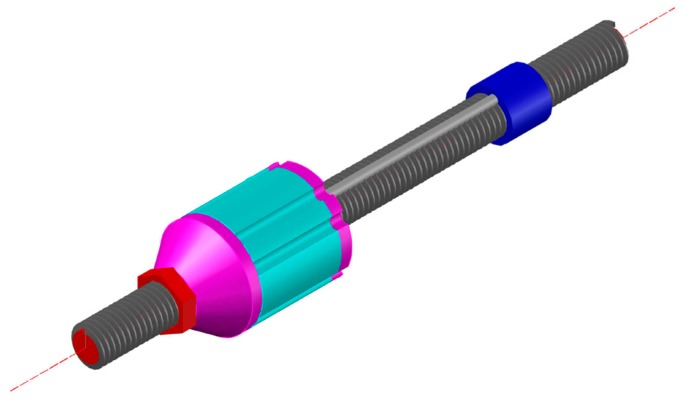
Photo of the Smart Rockbolt [72].

The Smart Rockbolt was proposed at Lulea University of Technology in Sweden [71]. The Smart Rockbolt is a standard rock bolt equipped with a strain sensor, accelerometer, processing module and wireless communication module. A photo of the Smart Rockbolt is shown in Figure 24. Integrated with the Internet of Things (IoT) technology, the Smart Rockbolt is able to continuously and simultaneously monitor the strain and vibration of the rock bolt. The Smart Rockbolt is under development and not yet commercially available.

**Figure 25:**
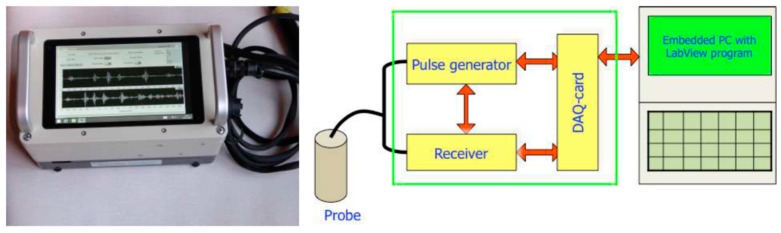
The portable, battery-supplied Rock Bolt Tester (RBT) instrument (**left**), and its simplified block diagram (**right**) [73].

A group at AGH University of Science and Technology in Poland devised a new Rock Bolt Tester (RBT) for rock bolt testing using guided ultrasonic waves [73]. The RBT is a portable instrument that consists of specially designed analog electronics for generating and receiving the guided waves and an embedded digital computer for signal processing, operator communication and data storage. The photograph and the block diagram of the RBT are shown in Figure 25. The RBT was used to determine the rock bolt condition, especially the grouting condition.

**Figure 26:**
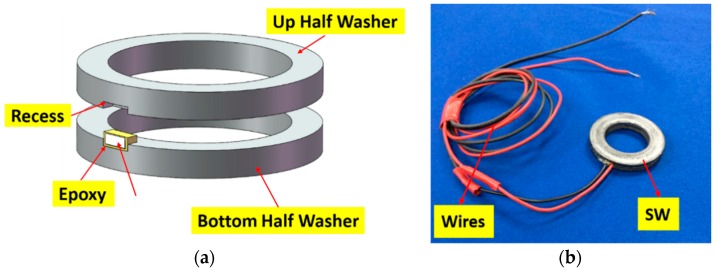
(**a**) The design of the smart washer; (**b**) Photograph of the smart washer [74].

**Figure 27:**
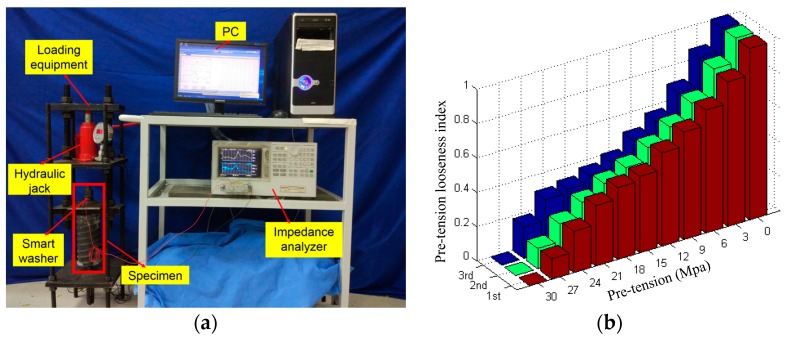
(**a**) The experimental setup; (**b**) the normalized RMSD-based rock bolt looseness indices of three repeating experiments [74].

More recently, Wang et al. proposed the Smart Washer for looseness of the rock bolt based on the electro-mechanical impedance method [74]. As shown in Figure 26, the Smart Washer was fabricated by sandwiching a piezoceramic transducer into two metal rings. The Smart Washer was used to monitor the looseness of the rock bolt based on the variations of the impedance signatures. The normalized root mean square deviation (RMSD) index was developed to evaluate the degradation level of the rock bolt pre-stress. Figure 27a shows the experimental setup and Figure 27b shows the RMSD indices corresponding to different loading conditions. As can be seen, the RMSD indices can effectively indicate the looseness level of the rock bolt.

## 5. Future Trends

From the above mentioned literature, rock bolt technology has taken a direction towards instrumentation with sensors. While rock bolts have been shown to greatly help stabilize rock formations and increase safety, knowledge of the bolt condition will be essential for the continued function of rock bolt systems over time. Depending on the parameter to be monitored, different types of sensors are used and installed at different locations of the rock bolt system. The interfaces in the rock bolt system and the loads experienced by the bolt are key areas that need monitoring. Specifically, the fiber optic sensors, and shear mode smart aggregates can be applied to assess the interface condition between the rock bolt and the grout, and the interface condition between the grout and the rock mass. The piezoelectric-based active sensing technique can also be used to monitor the bolt load, and bolt looseness [75,76]. Thus, the trend observed from the body of literature is that fiber optic sensors and piezoelectric transducers have taken a front seat in the innovation of rock bolt monitoring systems. 

A challenge in implementing rock bolt monitoring systems is robustness against the typically harsh and remote environment. With the cost of electronics becoming cheaper, different types of sensors, sensing interrogation systems, and communication systems will become more readily available for rock bolt monitoring and will overcome more and more of the challenge. Fiber optic sensors are useful in monitoring within areas with high electromagnetic interference and high temperatures. On the other hand, piezoelectric transducers can provide critical information that may not be gathered by fiber optic sensors. However, piezoelectric transducers require proper protection and obtaining signals from sensors can be problematic due to cabling issues. In the future, the introduction of radio frequency identification (RFID) may be used to obtain the overall information of the rock bolts installed in critical locations without the need for excessive cabling.

## 6. Conclusions

In this review paper, the basic principles of commonly used smart sensors used in rock bolt monitoring are briefly reviewed, followed by a description of the state-of-the-art applications of these smart sensors in monitoring the health condition of rock bolts. These smart sensors are the piezoceramic sensors and fiber optic sensors. Several aspects of rock bolt monitoring using these smart sensors are critically reviewed, including axial load monitoring, corrosion monitoring, grout monitoring and delamination monitoring. Lastly, the smart rock bolt concept was introduced in rock bolt monitoring and several smart rock bolt devices were reviewed. In conclusion, the application of smart sensors in rock bolt monitoring is becoming mature and they have replaced conventional sensors in various real world applications. With the capability of smart sensors, in-line and real-time condition monitoring of rock bolts can be achieved, which will provide more critical safety information on underground mining.

## References

[1] Benmokrane B., Chekired M., Xu H. (1995). Monitoring behavior of grouted anchors using vibrating-wire gauges. J. Geotech. Eng..

[2] Kılıc A., Yasar E., Atis C. (2003). Effect of bar shape on the pull-out capacity of fully-grouted rockbolts. Tunn. Undergr. Space Technol..

[3] Mark C., Pappas D., Barczak T. (2011). Current trends in reducing ground fall accidents in US coal mines. Min. Eng..

[4] Maiti J., Khanzode V.V. (2009). Development of a relative risk model for roof and side fall fatal accidents in underground coal mines in india. Saf. Sci..

[5] Buys B.J., Heyns P.S., Loveday P. (2009). Rock bolt condition monitoring using ultrasonic guided waves. J. South. Afr. Inst. Min. Metall..

[6] Duzgun H., Einstein H. (2004). Assessment and management of roof fall risks in underground coal mines. Saf. Sci..

[7] Crosky A., Smith B., Hebblewhite B. (2003). Failure of rockbolts in underground mines in australia. Pract. Fail. Anal..

[8] Kelly A., Jager A. (1996). Critically evaluate techniques for the in situ testing of steel tendon grouting effectiveness as a basis for reducing fall of ground injuries and fatalities.

[9] Li C.C. (2010). A new energy-absorbing bolt for rock support in high stress rock masses. Int. J. Rock Mech. Min. Sci..

[10] Kılıc A., Yasar E., Celik A. (2002). Effect of grout properties on the pull-out load capacity of fully grouted rock bolt. Tunn. Undergr. Space Technol..

[11] Canbulat I. (2008). Evaluation and Design of Optimum Support Systems in South African Collieries Using the Probabilistic Design Approach. Ph.D. Thesis.

[12] Hoek E., Kaiser P.K., Bawden W.F. (1995). Support of Underground Excavations in Hard Rock.

[13] Li C. Design principles of rock support for underground excavations. Proceedings of the ISRM International Symposium-EUROCK 2012.

[14] Luo J. (1999). A New Rock Bolt Design Criterion and Knowlwdge-Based Expert System for Stratified Roof. Ph.D. Thesis.

[15] Li C.C. (2012). Performance of d-bolts under static loading. Rock Mech. Rock Eng..

[16] Peng S.S., Tang D. (1984). Roof bolting in underground mining: A state-of-the-art review. Int. J. Min. Eng..

[17] Kendorski F.S. (2003). Rock reinforcement longevity. Geo-Strata—Geo Inst. ASCE.

[18] Tressler J.F., Alkoy S., Newnham R.E. (1998). Piezoelectric sensors and sensor materials. J. Electroceram..

[19] Duan W.H., Wang Q., Quek S.T. (2010). Applications of piezoelectric materials in structural health monitoring and repair: Selected research examples. Materials.

[20] Giurgiutiu V. (2007). Structural Health Monitoring: With Piezoelectric Wafer Active Sensors.

[21] Lin X., Yuan F. (2001). Diagnostic lamb waves in an integrated piezoelectric sensor/actuator plate: Analytical and experimental studies. Smart Mater. Struct..

[22] Kessler S.S., Spearing S.M., Soutis C. (2002). Damage detection in composite materials using lamb wave methods. Smart Mater. Struct..

[23] Giurgiutiu V. (2005). Tuned lamb wave excitation and detection with piezoelectric wafer active sensors for structural health monitoring. J. Intell. Mater. Syst. Struct..

[24] Liang Y., Li D., Parvasi S.M., Kong Q., Song G. (2016). Bond-slip detection of concrete-encased composite structure using electro-mechanical impedance technique. Smart Mater. Struct..

[25] Dugnani R., Zhuang Y., Kopsaftopoulos F., Chang F.-K. (2016). Adhesive bond-line degradation detection via a cross-correlation electromechanical impedance–based approach. Struct. Health Monit..

[26] Park S., Lee J.-J., Yun C.-B., Inman D.J. (2008). Electro-mechanical impedance-based wireless structural health monitoring using pca-data compression and k-means clustering algorithms. J. Intell. Mater. Syst. Struct..

[27] Chan H., Masserey B., Fromme P. (2015). High frequency guided ultrasonic waves for hidden fatigue crack growth monitoring in multi-layer model aerospace structures. Smart Mater. Struct..

[28] Li W., Kong Q., Ho S.C.M., Mo Y., Song G. (2016). Feasibility study of using smart aggregates as embedded acoustic emission sensors for health monitoring of concrete structures. Smart Mater. Struct..

[29] Perelli A., De Marchi L., Marzani A., Speciale N. (2012). Acoustic emission localization in plates with dispersion and reverberations using sparse pzt sensors in passive mode. Smart Mater. Struct..

[30] Beard M., Lowe M. (2003). Non-destructive testing of rock bolts using guided ultrasonic waves. Int. J. Rock Mech. Min. Sci..

[31] Beard M., Lowe M., Cawley P. (2003). Ultrasonic guided waves for inspection of grouted tendons and bolts. J. Mater. Civil Eng..

[32] Li H.-N., Li D.-S., Song G.-B. (2004). Recent applications of fiber optic sensors to health monitoring in civil engineering. Eng. Struct..

[33] Meltz G., Morey W.W., Glenn W. (1989). Formation of bragg gratings in optical fibers by a transverse holographic method. Opt. Lett..

[34] Lau K.-T., Yuan L., Zhou L.-M., Wu J., Woo C.-H. (2001). Strain monitoring in frp laminates and concrete beams using fbg sensors. Compos. Struct..

[35] Wu Z., Xu B., Hayashi K., Machida A. (2006). Distributed optic fiber sensing for a full-scale pc girder strengthened with prestressed pbo sheets. Eng. Struct..

[36] Zhang D., Shi B., Cui H., Xu H. (2004). Improvement of spatial resolution of brillouin optical time domain reflectometer using spectral decomposition. Opt. Appl..

[37] Barrias A., Casas J.R., Villalba S. (2016). A review of distributed optical fiber sensors for civil engineering applications. Sensors.

[38] Mitri H. Evaluation of rock support performance through instrumentation and monitoring of bolt axial load. Proceedings of the 11th Underground Coal Operators’ Conference.

[39] Mitri H., Marwan J. A new rockbolt axial load measuring device. Proceedings of the 20th International Conference on Ground Control in Mining.

[40] Mitri H., Laroche L. New technology for ground monitoring in underground mines using instrumented rockbolts. Proceedings of the Mine Planning and Equipment Selection Symposium.

[41] Frank A., Nellen P.M., Broennimann R., Sennhauser U.J. Fiber optic bragg grating sensors embedded in gfrp rockbolts. Proceedings of the 1999 Symposium on Smart Structures and Materials.

[42] Nellen P.M., Broennimann R., Frank A., Mauron P., Sennhauser U.J. Structurally embedded fiber bragg gratings: Civil engineering applications. Proceedings of the Photonics East’99, International Society for Optics and Photonics.

[43] Nellen P.M., Frank A., Broennimann R., Sennhauser U.J. Optical fiber bragg gratings for tunnel surveillance. Proceedings of the SPIE’s 7th Annual International Symposium on Smart Structures and Materials.

[44] Schroeck M., Ecke W., Graupner A. (2000). Strain monitoring in steel rock bolts using fbg sensor arrays. Proc. SPIE.

[45] Moerman W., Taerwe L., De Waele W., Degrieck J., Himpe J. (2005). Measuring ground anchor forces of a quay wall with bragg sensors. J. Struct. Eng..

[46] Ho S.C.M., Li W., Wang B., Song G. (2017). A Load Measuring Anchor Plate for Rock Bolt Using Fiber Optic Sensor. Smart Mater. Struct..

[47] Weng X., Ma H., Wang J. (2015). Stress monitoring for anchor rods system in subway tunnel using fbg technology. Adv. Mater. Sci. Eng..

[48] Do T.M., Kim Y.-S. (2016). Prediction of load transfer depth for cost-effective design of ground anchors using fbg sensors embedded tendon and numerical analysis. Geomech. Eng..

[49] Iten M., Puzrin A.M. (2010). Monitoring of stress distribution along a ground anchor using botda. Proc. SPIE.

[50] Moffat R.A., Beltran J.F., Herrera R. (2015). Applications of botdr fiber optics to the monitoring of underground structures. Geomech. Eng..

[51] Spearing A., Mondal K., Bylapudi G., Hirschi J. The corrosion of rock anchors in US coal mines. Proceedings of the SME Annual Meeting.

[52] Aziz N., Craig P., Nemcik J., Hai F. (2014). Rock bolt corrosion—An experimental study. Min. Technol..

[53] Craig P., Serkan S., Hagan P., Hebblewhite B., Vandermaat D., Crosky A., Elias E. (2016). Investigations into the corrosive environments contributing to premature failure of australian coal mine rock bolts. Int. J. Min. Sci. Technol..

[54] Vandermaat D., Saydam S., Hagan P., Crosky A. (2016). Examination of rockbolt stress corrosion cracking utilising full size rockbolts in a controlled mine environment. Int. J. Rock Mech. Min. Sci..

[55] Spearing A., Mondal K., Bylapudi G., Hirschi J.C. A method to determine the corrosion potential of rock bolts on coal mines. Proceedings of the 29th International Conference on Ground Contorl in Mining.

[56] Wei H., Zhao X., Li D., Zhang P., Sun C. (2015). Corrosion monitoring of rock bolt by using a low coherent fiber-optic interferometry. Opt. Laser Technol..

[57] Zou D.S. (2004). Analysis of in situ rock bolt loading status. Int. J. Rock Mech. Min. Sci..

[58] Madenga V., Zou D., Zhang C. (2006). Effects of curing time and frequency on ultrasonic wave velocity in grouted rock bolts. J. Appl. Geophys..

[59] Cui Y., Zou D. (2006). Numerical simulation of attenuation and group velocity of guided ultrasonic wave in grouted rock bolts. J. Appl. Geophys..

[60] Zou D.S., Cheng J., Yue R., Sun X. (2010). Grout quality and its impact on guided ultrasonic waves in grouted rock bolts. J. Appl. Geophys..

[61] Cui Y., Zou D. (2012). Assessing the effects of insufficient rebar and missing grout in grouted rock bolts using guided ultrasonic waves. J. Appl. Geophys..

[62] Zou D., Cui Y., Madenga V., Zhang C. (2007). Effects of frequency and grouted length on the behavior of guided ultrasonic waves in rock bolts. Int. J. Rock Mech. Min. Sci..

[63] Zou D., Cui Y. (2011). A new approach for field instrumentation in grouted rock bolt monitoring using guided ultrasonic waves. J. Appl. Geophys..

[64] Lee J., Kim H., Lee I., Han S., Lee Y. Rock bolt integrity evaluation in tunnelling using ultrasonic ndt techniques. Proceedings of the Proceedings of the World Tunnel Congress 2007 and 33rd ITA/AITES Annual General Assembly.

[65] Han S.-I., Lee I.-M., Lee Y.-J., Lee J.-S. (2009). Evaluation of rock bolt integrity using guided ultrasonic waves. Geotech. Test. J..

[66] Lee I.-M., Han S.-I., Kim H.-J., Yu J.-D., Min B.-K., Lee J.-S. (2012). Evaluation of rock bolt integrity using fourier and wavelet transforms. Tunn. Undergr. Space Technol..

[67] Yu J.-D., Bae M.-H., Lee I.-M., Lee J.-S. (2012). Nongrouted ratio evaluation of rock bolts by reflection of guided ultrasonic waves. J. Geotech. Geoenviron. Eng..

[68] He C., Van Velsor J., Lee C., Rose J. Health monitoring of rock bolts using ultrasonic guided waves. Proceedings of the AIP Conference Proceedings.

[69] Zeng L., Parvasi S.M., Kong Q., Huo L., Li M., Song G. (2015). Bond slip detection of concrete-encased composite structure using shear wave based active sensing approach. Smart Mater. Struct..

[70] Starkey A., Ivanovic A., Neilson R.D., Rodger A.A. (2003). Using a lumped parameter dynamic model of a rock bolt to produce training data for a neural network for diagnosis of real data. Meccanica.

[71] Delsing J., Eliasson J., Pereira P.P., Gebart J. The IoT rockbolt. Proceedings of the 5th International Conference on Internet of Things.

[72] Puñal Pereira P. (2016). Efficient IoT Framework for Industrial Applications. Ph.D. Thesis.

[73] Stepinski T., Matsson K.-J. Rock bolt inspection by means of rbt instrument. Proceedings of the 19th World Conference on Non-Destructive Testing.

[74] Wang B., Huo L., Chen D., Li W., Song G. (2017). Impedance-based pre-stress monitoring of rock bolts using a piezoceramic-based smart washer—A feasibility study. Sensors.

[75] Yin H., Wang T., Yang D., Liu S., Shao J., Li Y. (2016). A smart washer for bolt looseness monitoring based on piezoelectric active sensing method. Appl. Sci..

[76] Wang T., Song G., Wang Z., Li Y. (2013). Proof-of-concept study of monitoring bolt connection status using a piezoelectric based active sensing method. Smart Mater. Struct..

